# Progressing the development of a food literacy questionnaire using cognitive interviews

**DOI:** 10.1017/S1368980021004560

**Published:** 2022-07

**Authors:** Courtney Thompson, Jean Adams, Helen Anna Vidgen

**Affiliations:** 1 Queensland University of Technology (QUT), Faculty of Health, School of Exercise and Nutrition Sciences, Victoria Park Road, Kelvin Grove, QLD 4059, Australia; 2 Centre for Diet and Activity Research (CEDAR), MRC Epidemiology Unit, University of Cambridge School of Clinical Medicine, Cambridge, UK

**Keywords:** Food literacy, Survey, Qualitative methods, Think-aloud protocols, Thematic analysis

## Abstract

**Objective:**

Food literacy is the knowledge, skills and behaviours needed to meet food needs and determine intake and is conceptualised as eleven components under four domains of planning and managing, selecting, preparing, and eating. Previous measures of food literacy vary in their adherence to the conceptualisation and ability to capture totality of eating. This study aimed to determine items for inclusion and exclusion in a food literacy item pool and capture the general public’s interpretation of everyday food literacy practices.

**Design:**

Beginning with an item pool from previous studies, cognitive interviews were conducted using think-aloud and verbal probing methods. Data were first analysed for applicability, clarity, ambiguity and logic, then for emergent themes to ensure items captured the totality of the participant’s eating.

**Setting:**

Australia

**Participants:**

Australian residents over 18 years of age recruited via Facebook residential groups (*n* 20).

**Results:**

Of the original 116 items, 11 items had limited applicability; 13 items had unclear references; 32 items had lexical problems and 11 items had logical problems. In total, 29 items were deleted, 31 retained and 56 revised. Thematic analysis revealed participants limited their responses to consider only conventional practices such as grocery shopping, cooking and planned meals rather than the totality of their eating. An additional eighty-four items were developed to address eating out, incidental eating occasions and inconsistencies between participants assumed correct knowledge and that of public health guidelines. This resulted in a refined 171-item pool.

**Conclusions:**

This study progresses the development towards a comprehensive, validated food literacy questionnaire.

Food literacy has been defined as ‘… a collection of interrelated knowledge, skills and behaviours required to plan, manage, select, prepare and eat food to meet needs and determine intake’ and the ‘… scaffolding that empowers individuals, households, communities or nations to protect diet quality through change and strengthen dietary resilience over time’.^(1)^. This definition is supported by a conceptual framework, consisting of eleven components of food literacy organised into four interrelated domains of planning and managing, selecting, preparing, and eating (see Fig. 1). Although many definitions and frameworks of food literacy exist^(2–5)^, this is the first to be empirically derived. It is also proposed as the most current, predominant approach to food literacy that significantly advances the field^(2,4,6)^. However, many issues have been identified in the progression towards measurement of this construct. In a scoping review of food literacy tools, Amouzandeh *et al.* ^(7)^ identified twelve questionnaires designed to assess food literacy. While some level of validation had been conducted on most tools, none comprehensively adhered to the proposed framework (see Fig. 1) in capturing the four domains and eleven components of food literacy^(1)^.

**Fig. 1:**
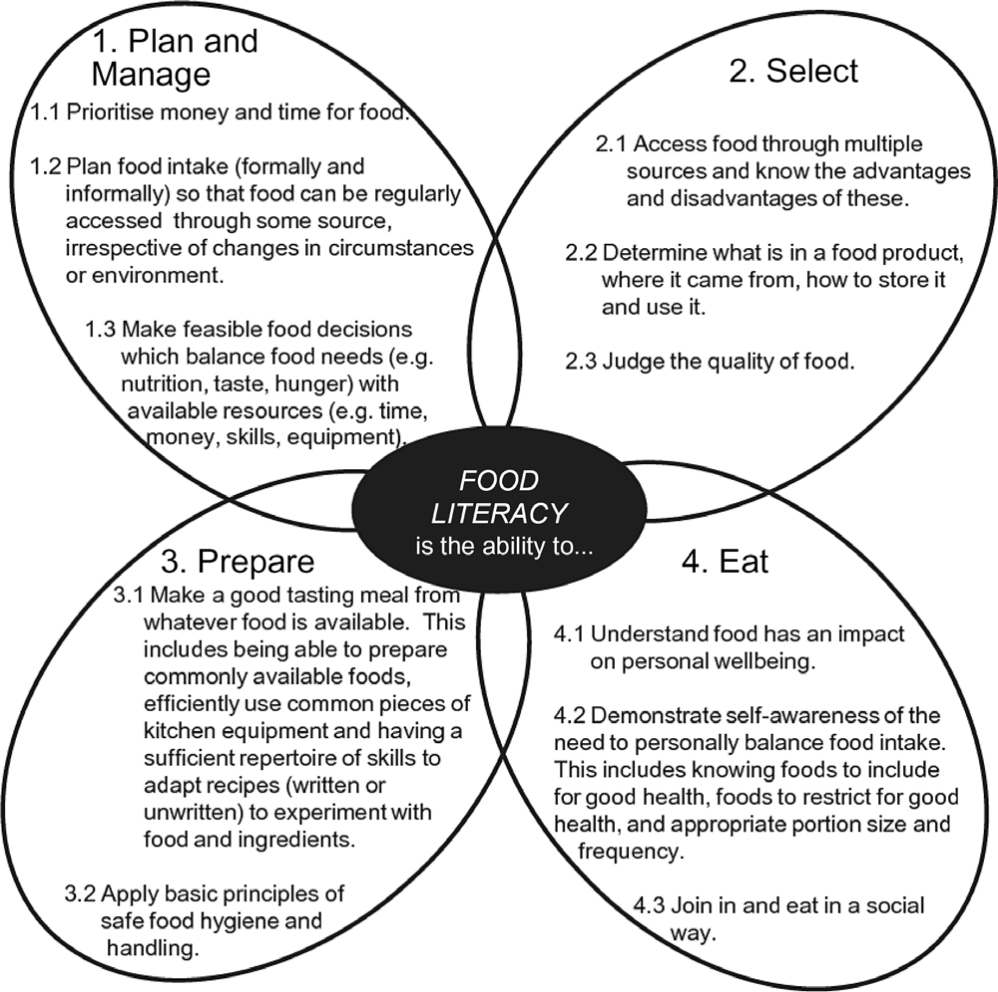
Domains and components of food literacy by Vidgen & Gallegos^(1)^

To progress measurement development, Fingland *et al.* ^(8)^ conducted a content validity study with 85 international experts across 20 countries to reach consensus on 151 previously validated items that reflected the Vidgen & Gallegos^(1)^ model in 2 rounds of feedback. Some consensus was achieved, with quantitative data resulting in 119 items retained. The next step to progress questionnaire development typically involves a face validity study to determine the general public’s understanding around wording and interpretability of food literacy items. However, when qualitative data was analysed, experts agreed on constructs relating to food preparation, grocery shopping, structured meals and less on the planning and selection involved with eating outside of the home. With growing research highlighting the significant contribution of food preparation and consumption outside of the home to total dietary intake^(9–13)^, it is integral that this construct is effectively captured. Thus, we considered the need to capture further information regarding the general public’s food literacy practices was critical in the development of a robust food literacy questionnaire. Building on existing work by Fingland *et al.* ^(8)^, the purpose of this study was to determine items for inclusion and exclusion in a food literacy item pool which reflected the four domains and eleven components of food literacy^(1)^. This involved conducting cognitive interviews with a sample of Australian adults, determining items to retain, revise or delete and thematically analysing responses to understand the general public’s interpretation of items relating to food literacy practices and ensure these were effectively captured.

## Methods

Cognitive interviewing, a method used to study understanding, mental processing and responses to material presented^(14)^, was chosen for two reasons. Firstly, cognitive interviews allow for participants to identify areas of complexity within questionnaire tools, report social desirability bias and ensure question items and response options are not misleading^(15)^. Secondly, as cognitive interviews are exploratory in nature, they provide rich insight into ideas, attitudes and understandings on the construct under study which may help to generate items that more effectively capture the construct^(16)^. Therefore, cognitive interviews extend beyond simple face validity techniques often employed in food literacy research (see online supplementary material, Supplemental Table S2) to determine which items are unclear and how they are unclear to guide questionnaire revisions. Conrad, Blair & Tracy^(17)^ and Willis^(18)^ report on two key procedures that characterise cognitive interview methods: think-aloud and verbal probing. These methods are commonly cited, often used in health research^(19–23)^ and more broadly consider the background social context that influences questionnaire items^(24,25)^. Think-aloud methods ask the participant to verbalise thinking as they answer the question^(26)^, while verbal probing involves the interviewer asking the participant probe questions to further elucidate thinking; these probes can relate to comprehension, interpretation, confidence, judgement or recall and can be scripted or unscripted^(18,27)^.

### Study participants and recruitment

We included participants who were residents of Australia, over 18 years of age, proficient in the English language and had not completed a nutrition or dietetic qualification^(28,29)^. Participants were recruited between the 26 May 2020 and 1 June 2020 via advertisements posted in eight residential Facebook groups in Queensland from lower (*n* 5), middle (*n* 2) and high (*n* 1) socio-economic index levels with approximately 426 000 total members.

A sample size of twenty participants were recruited as Blair *et al.* ^(30)^ found that in samples of 5–50 participants, the identification of new questionnaire problems slowed at this sample size, and were therefore closer to achieving data saturation^(31)^. Additionally, similar studies reported data saturation at fourteen and eighteen interviews, respectively^(32,33)^. Participants completed an online screening questionnaire via KeySurvey^(34)^ determining age, residency status, highest level of education and whether they had previously obtained a nutrition qualification. They also provided their contact details and availability over a 2-week period to participate in the interview. Eligible participants were emailed a calendar invite to attend a private, password protected video conference meeting via Zoom^(35)^ with the first author (C.T.). Zoom was chosen for the cognitive interviews due to the COVID-19 pandemic which prevented in-person data collection and to allow for a more geographically diverse sample. The following demographic data were also collected in addition to that obtained during screening in order to ensure representativeness of the sample: gender (target of ten males and ten females), main job (categorised using the Australian and New Zealand Standard Classification of Occupations^(36)^ if employed for payment or profit, and according to the Australian Bureau of Statistics Census categorisations if not^(37)^) and postcode (to determine socio-economic advantage and disadvantage) using SEIFA^(38)^.

### Item pool

The development of the pool for testing began with the inclusion of items which had achieved consensus in a related content validity study by Fingland *et al.* (2020)^(8)^. Quantitative feedback, in the form of Likert scale content relevance resulted in 119 items being retained. Qualitative feedback, in the form of open-ended comments on these 119 items were re-analysed for issues relating to readability, wording and understanding, resulting in 15 items excluded, 75 items rephrased and 12 new items developed. Overall, 116 items were included in the preliminary food literacy questionnaire assessed in this study (see online supplementary material, Supplemental Table S1). The Flesch Reading Ease test score was 68·0 (on a 100-point scale, with optimum scoring between 60 and 70)^(39)^ and the Flesch–Kincaid Grade Level test, determined using average sentence length and average number of syllables per word across a document, indicative of the grade level the text can be understood at, was 6·1^(40)^.

### Cognitive interview methods

Think-aloud interviewing protocols^(25)^ were used for the cognitive interviews, whereby participants were asked to read the item from the food literacy pool out-loud from a PowerPoint^(41)^ slide and verbalise their thoughts as they answered the question. Verbal cognitive probing was used to elicit more detailed information on how participants understood and made judgements about items^(19)^. Table 1 presents an example of how the Willis & Artino^(25)^ ‘think-aloud’ interview method was applied. Additionally, unscripted probes were used based on respondents behaviour during the interview (e.g. frowning, hesitation, re-reading of items and pausing) to identify question similarity, language and problem-solving processes^(20)^.

**Table 1:** Verbal probes used in food literacy cognitive interviews^(25,27)^

Type of cognitive probe	Example: Q3, Component 1.1 (I try to purchase a variety of different types of food, even if it costs more)
Comprehension, interpretation	What does the term ‘variety’ mean to you?
Paraphrasing	Can you repeat the question in your own words?
Confidence judgement	How sure are you that you purchase a variety of different types of food, even if it costs more?
Recall	How do you remember that you purchase a variety of foods? How did you come up with your answer?
Specific	Why do you say you think that you purchase a variety of foods even if it costs more?
General	Was that easy or hard to answer? I noticed that you hesitated; tell me what you were thinking? Could you tell me more about that?

### Data collection

On attending the video conference, participants were provided information about the study, able to ask further questions about participation and given the opportunity to opt out or provide written signed consent via KeySurvey. Eighteen (90 %) of participants had cameras on during the interviews and two (10 %) asked to keep cameras off. Participants were allocated using a random number generator to respond to items from two of the four domains of food literacy; therefore, all domains and components were reviewed ten times. At the end of each component, the food literacy framework was displayed and participants were asked to comment on whether the items they just reviewed assessed the component and if not, provide suggestions for alternate items. Participants received a $15 e-voucher for participation.

### Data analysis

Interviews were audio- and video-recorded and auto transcribed using the transcription function in video conference software, Zoom, version 5.3.1. The interviewer (C.T.) checked and edited all transcripts for accuracy. The completed transcripts were imported into NVivo 12^(42)^ for analysis. Nodes for each item under the domains and components were developed, and participant responses to each item were highlighted and categorised into the appropriate node.

Data was analysed using a combination of two methods proposed by Conrad & Blair^(43)^ and Knafl *et al.* ^(44)^. The resulting coding scheme consisting of four categories and corresponding notes in NVivo were created. This comprised (a) limited applicability, defined as comments noting groups or situations for which the item would not be appropriate^(44)^, (b) unclear reference, defined as a lack of clarity regarding what situation the item is intended to address^(44)^, (c) lexical problems, defined as comments about meaning of words or terms that are confusing or ambiguous^(43)^ and (d) logical problems, defined as items which captured the same information in previous sections^(43)^. All responses were coded by two researchers, C.T. and H.V., independently and discussed if there was disagreement.

Secondly, the number of issues for each item identified from the coding scheme were then used to inform whether items would be retained, revised or deleted in line with similar methods reported by Knafl *et al.* ^(44)^. If only one person commented on an issue with an item, the item was retained as it was. If the problems identified could be addressed by re-wording, the question was revised. If there were multiple, competing interpretations of the item or substantial problems that could not be addressed by rewording, the item was deleted.

C.T. and H.V. thematically analysed responses, and differences in coding scheme allocations to items were discussed until agreement was reached. Any revisions to existing items or development of new items by the two authors were reviewed by J.A.

## Results

### Participant characteristics

The screening survey was opened 733 times. One-hundred and twenty-five people (17 %) partially completed the screening questionnaire, with forty-five exiting prior to providing their name and email address (36 %) and eighty exiting prior to providing availability for the interview (64 %). Overall, sixty people completed the screening questionnaire (8 %). Of these, thirty-one people were contacted to participate in an interview (52 %); of whom nine did not respond to the initial email or the follow-up email inviting them to participate (29 %) and two people agreed to participate but did not attend the interview (6 %). Overall, twenty people participated, for whom the demographics are summarised in Table 2.

**Table 2:** Demographics characteristics of study participants (*n* 20)

	Study participants (*n* 20)	%
Gender		
Male	09	45
Female	11	55
Age		
20–29	11	55
30–39	01	05
40–49	02	10
50–59	02	10
60–69	03	15
70–79	01	05
Highest level of education		
Secondary/high school equivalent	05	25
Trade or other certificate	04	20
Diploma	07	35
Degree	04	20
Postgraduate degree	00	00
Job classifications*		
Managers	04	20
Professionals	02	10
Technicians and trades workers	02	10
Community and personal service workers	04	20
Clerical and administrative workers	02	10
Sale workers	01	05
Not in the workforce (student and retired)	03	15
Socio-economic advantage and disadvantage†		
1 (most disadvantaged)	01	05
2	00	00
3	00	00
4	00	00
5	00	00
6	02	20
7	01	05
8	01	05
9	04	20
10 (most advantaged)	11	55

* Determined using the ABS Australian & New Zealand Standard Classification of Occupations, 2013^(58)^.

† Australian Bureau of Statistics (ABS) postal area code by indexes for Australia (SEIFA)^(59)^. An area with a high score on this index has a relatively high incidence of advantage and a relatively low incidence of disadvantage.

### Classifying and responding to issues identified in participant comments

The average interview time was 48 min (range 31–97 min). Overall, 58 out of the 116 items (50 %) were identified as having one of the 4 categories of issues; 115 participant comments relating to items were reported, 49 % of which related to lexical problems (Table 3). Component 2.2 ‘Determine what is in a food product, where it came from, how to store it and use it’ was identified as having the most participants comments, and most comments per items (20, Table 3), while 1.3 ‘Make feasible food decisions which balance food needs with available resources’ and 4.2 ‘Demonstrate self-awareness of the need to personally balance food intake. This includes knowing foods to include for good health, foods to restrict for good health and appropriate portion size and frequency’ had the least (3, Table 3). Overall, thirty-one items were categorised as retain, fifty-six as revise and twenty-nine as delete.

**Table 3:** Distribution of participant comments across items in the eleven components of food literacy and four categories of reporting issues

			Limited applicability		Limited applicability		Limited applicability		Limited applicability		Mean comments per item
Component	No. items	No. total comments	No. comments	%	No. comments	%	No. comments	%	No. comments	%	
1.1	08	013	07	54	03	23	03 0	23	00	54	1.6
1.2	07	012	00	54	11	92	00000		01 0	8	1.7
1.3	09	003	00	54	00	54	03	100	00	54	0.3
2.1	09	016	02	13	02	13	070	44	05	30	1.8
2.2	10	020	02	10	00	54	130	54	05	54	2.0
2.3	07	007	00	54	02	54	030	54	02	54	1.0
3.1	20	009	02	22	01	11	040	45	02	22	0.5
3.2	13	011	03	27	00	54	080	73	00	54	0.8
4.1	07	010	00	54	00	54	10	100	00	54	1.4
4.2	17	005	00	54	00	54	05	100	00	54	0.3
4.3	09	009	04	44	04	44	00000		01	12	1.0
All (116)		115	20	17	23	20	560	49	16	14	1.0

#### Limited applicability

Eleven of the 116 items were identified by participants as not being applicable to all situations. Issues were identified relating to component 1.1, 2.1, 2.2, 3.1, 3.2 and 4.3 (see Table 3). Overall, items described typical living or food provisioning contexts that did not apply to all participants or were compound items. Examples of each are described below.

Reflected different living contexts: for Item 3_4.3, ‘In my household, people often eat at different times’, some participants responded stating that ‘…it doesn’t apply because yeah I live on my own, so I can’t really give an answer for that one I’m afraid.’ (Participant 3) and ‘I’m in a funny household at the moment… some people live alone.’ (Participant 15). The same issues were identified with item 6_4.3, ‘I often eat together with other people’.

Reflected different food provisioning contexts: In item 3_1.1, ‘I try to purchase a variety of different food even if it costs more’, a participant stated they, ‘… don’t do the buying… if you’re not the primary purchaser of the food then…I don’t know.’ (Participant 7).

Compound questions: In item 1_3.2, ‘I wash or peel fruit and vegetables before eating them’ participants stated ‘…I don’t peel all fruit and vegetables before I eat them? But I do wash everything before I eat them.’ (Participant 14) and ‘I agree to a certain extent with that, yes, I wash them. But I don’t always peel fruit or vegetables before eating them…’ (Participant 20).

To broadly address these issues, an answer option, ‘I don’t live with other people/I live alone’ was included to capture varying household situations, response options endpoints were changed from a strongly disagree to strongly agree to a never to always and compound questions were either split into two items or deleted to only address one part, based on the aspects participants believed the item should focus on (e.g. ‘I wash fruit and vegetables before eating them’).

#### Unclear reference

Thirteen of the 116 items were identified by participants as requiring clarification. Issues were identified most with component 1.2 (five comments), as well as 1.1, 2.1, 2.3, 3.1 and 4.3 (see Table 3). Overall, items needed clarification because they reflected unclear contexts, typical food purchasing, food provisioning or food socialisation behaviours that differed among participants or were vague. Examples of each are described below.

Clarity around location of food purchasing: In item 5_2.1, ‘I find the foods I prefer to eat’, participants commonly thought of the grocery store, opposed to other food sourcing occasions. For example, ‘So, is this like a grocery store or just anywhere?’ (Participant 8). Comparably, with item 6_2.1, ‘When I’m in a new place, I find the foods I prefer to eat’, participants needed clarification but tended not to consider the grocery store ‘So, does that mean eating out or?’ (Participant 15).

Clarity around food provisioning: In item 5_1.2, ‘I often prepare meals in advance to be eaten outside the home’ (1.2), participants were unsure of the setting: ‘So, I’m assuming this means if we’re heading out for, mainly for work or if we’re going for a picnic or something, I guess? Or if my kids are going to school?’ (Participant 6) and: ‘I would agree, strongly with that, if that’s referring to me going to work?’ (Participant 7).

Clarity around eating settings: The intent of item 7_4.3, ‘If I’m with other people, it is of great value to me to eat together’ was to determine the centrality of food in social settings, though this was not well understood: ‘So, sorry, does that mean if like, I’m with other people and I’m the only one eating or like if…’ (Participant 8) and: ‘With this one is this meaning like, if I’m with other people, we all eat together or kind of like, yeah?’ (Participant 19).

Clarity around definitions of a term: In item 1_1.2, ‘I often plan meals ahead’, timeframes were not included and participants sought clarification on this: ‘Ah… when you say ahead, like are we talking like a couple of days or are we talking like weeks?’ (Participant 3). ‘Okay. But how far ahead? I often plan meals ahead… so what do you, what’s your idea on ahead, a week ahead? Two days ahead?’ (Participant 10).

To broadly address these issues, occasions were more explicitly defined, for example, grocery store or a restaurant, work or place of study, day or week, while food socialisation was more explicitly described in terms of food centrality and relationships, for example, ‘Food is a central part of how I make friends or form relationships with other people’.

#### Lexical problems

Thirty-two of the 116 items were identified by participants as problematic or difficult to understand due to wording that was confusing or ambiguous. Issues were identified most with component 2.2 (six comments), alongside 1.1, 1.3, 2.1, 2.3, 3.1, 3.2, 4.1 and 4.2 (see Table 3). Overall, the items needed revising because specific terms used were confusing or ambiguous and needed defining. Examples of each are described below.

In item 4_2.2, ‘When eating out, I can make a judgement on what’s in the food I’ve selected’, participants needed clarification on the term ‘judgement’: ‘Does that mean like the nutritional information or the ingredients?’ (Participant 8) and: ‘…Are you asking about, can you identify what’s in the food you’ve selected from the menu or what you’re being told or you’re just making a judgement on what you think is in it?’ (Participant 20).

In item 4_4.1, ‘The type of food I eat influences whether I will experience particular illnesses’, participants were unsure what ‘particular illnesses’ meant: ‘Do you mean like cold and flu illnesses or like cancer illnesses?’ (Participant 9) and: ‘I don’t, particularly have many allergies or anything like that to foods, which is great… Is that the sort of illnesses you mean?’ (Participant 15).

Item 8_3.2, ‘I always store meats and dairy at low temperatures’ needed clarification by participants with regards to the meaning of ‘low temperatures’, for example: ‘Well, I think my fridge is at a low temperature. I don’t know what, maybe you need to specify what low temperatures are?’ (Participant 4) and: ‘…I think I assumed you meant like, that most people already keep them in the fridge but you want to keep them colder than a fridge.’ (Participant 9).

To broadly address these issues, terms identified as unclear were more clearly defined. For example, ‘When eating out, I can make a judgement on the nutritional value of the food I’ve selected’, ‘Eating foods high in saturated fat increases your risk of CVD’ and ‘To prevent food poisoning, your refrigerator temperature should be at or below 4 degrees Celsius’.

#### Logical problems

Eleven of the 116 items were identified by participants as having similarities to or overlapping with other items. Issues were identified relating to component 1.2, 2.1, 2.2, 2.3, 3.1 and 4.3 (see Table 3). Overall, items were similar because participants perceived them to be addressing the same constructs. Examples of each are described below.

In item 9_2.1, ‘When I’m in a new place, I find the foods that align with my values’ participants stated it was similar to a previous item, 7_2.1, ‘I find the foods that align with my values’: ‘I think this is kind of similar to the other question…’ (Participant 3) and: ‘It’s similar to a previous question… So, whether that was a new place, new restaurant, new, you know, it would be a similar thing.’ (Participant 20).

Item 10_2.2, ‘I know what’s in food I could buy if I’m eating out’ was identified as having similarities to previous items. One participant commented similarities to item 4_2.2, ‘When eating out, I can make a judgement on what’s in the food I’ve selected’: ‘Yeah, just, I strongly agree, based on what we’ve talked about in previous items.’ (Participant 15).

In item 16_3.1, ‘I have the skills to prepare and cook the foods I prefer’ participants commented on the similarity between this item and item 4_3.1 ‘I have the skills to prepare and cook affordable foods that I prefer’. This included feedback such as: ‘I think we went through this one didn’t we?’ (Participant 7) and ‘…I feel like, I feel like there was a similar question.’ (Participant 9).

To address these issues, one of the items that was identified as being similar was deleted; in this case, 9_2.1, 10_2.2 and 16_3.1.

### Thematic analysis of food literacy practices across domains

Analysis of item responses across the domains revealed participants limited their responses to particular food preparation or purchasing scenarios, such as cooking at home and grocery shopping, rather than the totality of their eating which included pre-prepared meals and takeaway or those consumed outside of the home at restaurants or cafés. Eleven examples are described below.

#### Planning and managing

Most items in domain 1, ‘planning and managing’, were interpreted in the context of food preparation in the home environment, for example, 1_1.2 ‘I often plan meals ahead’: ‘…it so heavily depends on how much I feel like cooking, it could be, you know, a case of I only have the mental capacity to make a sausage sizzle that day…’ (Participant 2), ‘…(if) I’ve got a heavy week of assignments and work I will, you know, that’s when I will do like, a massive spag bol…otherwise if I know I’ve got like a really slow week I like to then take the time to cook a good, you know, really flavoured full meal.’ (Participant 3), ‘… I certainly have an idea for the week. I know this week we’re having pumpkin soup one night, we’re having, I made a Japanese stir fry last night.’ (Participant 6).

Additionally, items related to food purchasing were always described in the context of grocery shopping. In item 2_1.3 ‘I often compare prices before I buy food’: ‘'I’ll just use milk straight up. You know you look at the price per litre. I have my own brand of milk, I buy Norco, but like if I was, you’d look at litre per price, like so say it’s like $1·10 per litre and the other one might be $1, I don’t know, $1·80 per litre. I’d look at that price difference.’ (Participant 3) and ‘If it’s like, for example, rice, I don’t really care. I just buy home brand because it’s just rice, like it tastes the same? But some things, like for example, chocolate, I’d rather buy Cadbury then home brand.’ (Participant 12). Similarly, in item 3_1.3 ‘I try to get the best food for the best price’, ‘100 % agree, yep price is a big thing. I just think about meat… that’s probably the biggest competitor, let’s be honest, you know, meat prices will vary so heavily.’ (Participant 3) and: ‘(My partner) loves pickles. He will go for what he thinks is tasty… even though it’s more expensive than the home brand ones.’ (Participant 20).

However, when it came to item 1_1.3 ‘I know how much money I spend on food in an average week’, one participant did describe budgeting in relation to eating out ‘Yeah, I’d agree on that one. I roughly, like in a normal week when I’m at uni, I’d probably spend a bit more money ‘cos sometimes I do take-out at uni. But I roughly know how much I spend.’ (Participant 12).

#### Selecting

In domain 2, ‘selecting’, constructs such as environmental or ethical impacts of foods were not well understood. For example, item 2_2.1 ‘I consider the environmental impacts of the foods I eat’ was always considered in the context of caged eggs and red meat, not processed foods or food delivery services^(45)^, ‘I do, because I did consider being a vegan at one stage because of the environmental impact, particularly of meat.’ (Participant 17) and: ‘I always trying to buy free range chicken and free-range eggs.’ (Participant 11).

There was more consideration of different eating occasions in this domain, for example, item 5_2.1 ‘I find the foods I prefer to eat’, ‘So, is this like at the grocery store?’ (Participant 8) and ‘I’m sort of looking at that thinking like when I’m eating out and also in the supermarket, markets.’ (Participant 17) and item 6_2.1 ‘When I’m in a new place, I find the foods I prefer to eat’, ‘I guess with the question when I’m in any place I’ll like really peruse the menu and make sure that I find something that I know I will like?’ (Participant 1), ‘I’m thinking of a restaurant or café. When I’m thinking twice about that, it could be at someone’s house as well.’ (Participant 11) and ‘I kind of thought of, like, a new like neighbourhood with like a shopping centre kind of area.’ (Participant 8).

Additionally, in item 2_2.3 ‘I can predict what processed or convenience foods will be like before I buy it’, participants considered different settings for obtaining these foods: ‘I’d say neutral and I kind of think of this one like fast foods, like there’s such a variance in like… like quality, I guess, of food.’ (Participant 1). ‘Not always. If you, it depends on the packaging they’re in. So if they’ve got some kind of coloured packaging, you can’t see through, you can’t see.’ (Participant 5).

#### Eating

In domain 4, ‘eating’, items on knowing foods to eat and restrict for good health were answered incorrectly when considering information in the Australian Dietary Guidelines^(46)^ and supporting documents^(47)^, for example, in item 6_4.2, ‘Processed meats, I’d put that in enjoy. I’m a little bit not great on that, but again, I see it as a bit of protein coming in.’ (Participant 3) and ‘Milk yogurt and cheese, I think you need to limit.’ (Participant 11).

Further, items about the nutritional content of foods were interpreted in the context of the grocery store, for example 2_4.2 ‘The nutritional content of food products is important to me when deciding what foods to buy’. ‘So I think of that as like the micronutrients on the back of like a box of cereal or something.’ (Participant 1), ‘I’m not going to buy these sugary muesli bars for my kids or juice boxes or poppers or whatever you call them ‘cos it’s just all full of sugar, they should just be drinking water.’ (Participant 11) and ‘I do now read labels, I particularly look at the, the sugars and the salts. I’m amazed at how much salt is in some preparations.’ (Participant 14). However, in the context of social eating, most interpreted this to mean outside of the home, for example, item 2_4.3: ‘Eating with other people is about more than just food’. ‘I would say I agree with that because usually we like, go out for coffee or a meal is like, kind of a social event.’ (Participant 8). ‘I find food a great way of bringing people together and starting conversations… inviting someone over for dinner or going out for dinner with someone is like, a reason to get together.’ (Participant 9).

#### Additional items

To address the issues described above, where there was confusion or responses varied among participants relating to the location of planning, selecting, preparing or consuming food outside of the home, five items were split to preface the occasion, for example, ‘When food shopping I…’ and ‘When buying from a restaurant, café or takeaway I…’.

Participants also gave feedback about whether items had comprehensively addressed the component of the Vidgen & Gallegos model^(1)^ they were reviewing (see Fig. 1 for component names).

For component 1.1: ‘… the thing that you’re trying to gauge is prioritising money and time for food, but I didn’t see any questions about if you are low on certain money items as a follow up question, what kinds of food do you prioritise if you only have enough money for one out of three or something.’ (Participant 1).

In component 1.3: ‘Yeah, I didn’t see too many questions regarding hunger in the actual questionnaire, but in the heading it does say that hunger would be a thing. But if you’re in a situation where you’re more hungry than you have time to prepare food, how do you decide whether or not to cook or what do you make instead.’ (Participant 1).

In component 2.2: ‘I think a way to incorporate the storage of food is, you know, do people know how the food is stored before it gets on the shelves… like, do they know how that (fresh foods) transported or how it goes from farm to shelf. And if they did know, would it change their minds about the choices that they make of which foods to buy.’ (Participant 13).

For component 3.2: ‘Maybe, maybe you could’ve ambushed someone with what the specific safe food temperatures for food.’ (Participant 16).

In component 4.2: ‘That’s the only thing, is do I know what proper portion sizes of are for each, category thing? I mean like, the recommended red meat intake is a lot less than what we eat. So, things like knowing if you were to have a portion of red meat, what size steak is that?’ (Participant 9).

Overall, eighty-four additional items were added. Forty items were added due to splitting existing items (seven resulting from prefacing the occasion, thirty-three added based on thematic analysis), while forty-four new questions were developed based on participant feedback relating to comprehensively addressing the components.

## Discussion

The purpose of this study was to progress the work conducted by Amouzandeh *et al.* ^(7)^ and Fingland *et al.* ^(8)^ to develop a food literacy questionnaire. This study refined an item pool that reflects the four domains and eleven components of food literacy described by Vidgen & Gallegos^(1)^ and provided insight into food literacy practices of Australian adults.

### Cognitive interviews and food literacy practices

Cognitive interviews effectively identified complexities with items in the original item pool. Only thirty-one items were retained as they were, with most being revised or deleted due to feedback from general public participants (73 %). This was unexpected, as all items in the pool were from tools that were described as being previously validated. However, on more detailed review we identified that only 22 of the 66 tools (33 %) where items were sourced from conducted interviews with the general public to assess understanding and interpretability of items: only one of which described using cognitive interview methods with adults (see online supplementary material, Supplemental Table S2). While conducting a face validity study provides some insight into items, this study found that while participants understood items, their interpretation varied from the intended purpose of the question by researchers. Items in component 4.1 and 4.2 on knowing foods to eat and restrict for good health were answered as something participants could do; however, further conversations resulted in responses that were not consistent with public health nutrition guidelines in Australia^(46,48)^. This highlights the value of conducting cognitive interviews to determine if items are interpreted and consistently responded to, particularly around conceptually challenging constructs.

Overall, participants largely understood the concept of food literacy, evident through their responses and recommendations for items that would more comprehensively address the components of food literacy. Additionally, thematic analysis of participants’ responses highlights that items in the food literacy questionnaire only captured food literacy practices and eating occasions such as grocery shopping and eating at home as opposed to purchasing pre-prepared foods and eating out at restaurants or cafes. This is problematic as it is likely to misrepresent an individual’s food literacy. Participant feedback and recommendations at this stage were integral in building on existing items to specify the context or occasion. Understanding people’s food literacy practices is critical as inclusion of recommendations in dietary guidelines and other public health nutrition advice is increasing in prevalence^(49)^; in turn, items that contribute to monitoring and surveillance that supports this is needed. Therefore, ensuring items are consistently interpreted and understood is important to the progression of future research in this field.

Thematic analysis of responses was more effective at identifying participant issues with items compared to quantitatively considering the number of issues identified with items. While this was a useful guide to determining if items would be retained, revised or deleted, issues with knowledge or context would not have been identified without this further analysis. Participant feedback on each component was integral in developing items to replace those recommended for deletion and ensured all eleven components of food literacy were comprehensively addressed. Finally, this step of conducting cognitive interviews was critical as experts are not able to determine if items are well understood in comparison to the general public.

### Strengths and limitations

The strengths of this research include the use of well-established methodological approaches to cognitive interviewing, conducting thematic analysis and random allocation of participants to domains and components of food literacy. As food literacy encompasses everyday practicalities involving food^(50)^, participants tended to describe how they enacted these behaviours and not how they reached their responses. As a result, using varying cognitive interviewing techniques were useful, specifically verbal probing, which was effective in re-focusing participants to describe how they reached their response and identify issues with the items^(51)^. Additionally, thematic analysis of participants’ responses was integral in developing items that better captured food literacy practices and ensured the domains and components of food literacy were comprehensively addressed. Finally, prior to the commencement of interviews, some participants indicated they did not feel particularly knowledgeable about the domain they were allocated. This may, however, have been advantageous as having poorer perceived knowledge may have resulted in items that were more comprehensively reviewed for clarity and understanding.

Limitations of this research include the online nature of the cognitive interviews and generalisability. All interviews were conducted online due to the COVID-19 pandemic. As some participants asked to keep their cameras off, unscripted probes may have been missed during these interviews. However, these were only visual (e.g. frowning) as hesitation, re-reading of items and pausing were still easily detectable via audio only. The cognitive interviews were conducted in one round, when often cognitive interviews are conducted in phases, so item issues can be addressed and approved by general public participants^(52)^. As items were added to the questionnaire resulting from participant feedback, additional interview phases may have been beneficial. However, for pragmatic reasons, this was not considered necessary as most participants provided suggestions to revise the wording of items prior to inclusion in the refined item pool. Despite advertisements in lower socio-economic residential Facebook groups, most participants were from higher socio-economic residential groups. This may have been due to Facebook algorithms, timing of advertisements or advertisement presentations were not engaging across the socio-economic spectrum^(53,54)^. Therefore, results may not be generalisable to all Australians or those from socio-economically diverse backgrounds. However, items had appropriate reading ease and grade-level scores, both of which improved further in refined item pool (Flesch Reading East test score = 64·9 and Flesch–Kincaid Grade Level = 4·8) indicating items could be understood by a broader range of the general public^(55,56)^. Additionally, further validation and reliability testing of the questionnaire will be conducted using market research panels, to specifically ensure those from lower socio-economic backgrounds are represented. Finally, there may be self-selection bias as participants who responded to the advert indicated they chose to participate due to an interest in food and nutrition^(57)^; however, this was more often mentioned by participants who identified as female, only half of the sample (55 %). Overall, accessing Facebook groups ahead of time to determine optimum posting windows, increased recruitment time and interview slots may address the limitations outlined.

## Conclusion

Cognitive interviews provided insight into participants understanding and interpretation of items, resulting in a pool of 171 items that comprehensively assess all domains and components of the construct as described by Vidgen & Gallegos^(1)^. This study progresses the development towards a comprehensive, validated food literacy questionnaire. Future research includes progressing the development of a food literacy questionnaire by evaluating the psychometric properties, factor structure and reliability of the refined item pool with a diverse sample of adults for gender, age and socio-economic status.

## Supplementary material

For supplementary material accompanying this paper visit https://doi.org/10.1017/S1368980021004560. click here to view supplementary material
