# Supplementary material for: Progressing the development of a food literacy questionnaire using cognitive interviews
Source: Public Health Nutr. 2021 Nov 8;25(7):1968–78. doi: 10.1017/S1368980021004560 (PMC9991617; doi:10.1017/S1368980021004560)
Supplement: Supplementary file 1 [file S1368980021004560sup.zip › S1368980021004560sup001.docx]

# **Supplementary Material**

## Table S1 Food literacy initial item pool

| **Domain 1: Plan and Manage (24 items)** | |
| --- | --- |
| **Component 1.1 Prioritise time and money for food** | |
| 1. Compared to other daily tasks, food shopping takes up too much of my time | a. Strongly disagree  b. Disagree  c. Neutral  d. Agree  e. Strongly agree |
| 1. Compared to other daily tasks, cooking or preparing food takes up too much of my time | a. Strongly disagree  b. Disagree  c. Neutral  d. Agree  e. Strongly agree |
| 1. I try to purchase a variety of different types of food even if it costs more | a. Strongly disagree  b. Disagree  c. Neutral  d. Agree  e. Strongly agree |
| 1. I know how to allocate money so I always have some money for food | a. Strongly disagree  b. Disagree  c. Neutral  d. Agree  e. Strongly agree |
| 1. Compared to other daily tasks, planning what food to buy or eat takes up too much of my time | a. Strongly disagree  b. Disagree  c. Neutral  d. Agree  e. Strongly agree |
| 1. I often eat food I’d prefer not to because I’ve run out of time to buy or prepare the food I’d prefer | a. Strongly disagree  b. Disagree  c. Neutral  d. Agree  e. Strongly agree |
| 1. I always try to have enough money set aside to feed the people I’m responsible for | a. Strongly disagree  b. Disagree  c. Neutral  d. Agree  e. Strongly agree |
| 1. Compared to other daily tasks, eating takes up too much of my time | a. Strongly disagree  b. Disagree  c. Neutral  d. Agree  e. Strongly agree |
| **Component 1.2 Plan food intake (formally and informally) so that food can be regularly accessed through some source, irrespective of changes in circumstances or environment** | |
| 1. I often plan meals ahead | a. Strongly disagree  b. Disagree  c. Neutral  d. Agree  e. Strongly agree |
| 1. I always plan what to buy when I shop for food | a. Strongly disagree  b. Disagree  c. Neutral  d. Agree  e. Strongly agree |
| 1. I am able to adapt my plans for what to eat even if my living circumstances change | a. Strongly disagree  b. Disagree  c. Neutral  d. Agree  e. Strongly agree |
| 1. When I go out, I think about whether I should take food with me | a. Strongly disagree  b. Disagree  c. Neutral  d. Agree  e. Strongly agree |
| 1. I often prepare meals in advance to be eaten outside the home | a. Strongly disagree  b. Disagree  c. Neutral  d. Agree  e. Strongly agree |
| 1. I often shop with specific meals in mind | a. Strongly disagree  b. Disagree  c. Neutral  d. Agree  e. Strongly agree |
| 1. I am able to adapt my plans for what to eat, even if something unexpected happens in the short term | a. Strongly disagree  b. Disagree  c. Neutral  d. Agree  e. Strongly agree |
| **Component 1.3 Make feasible food decisions which balance food needs (e.g. nutrition, taste, hunger) with available resources (time, money, skills, equipment)** | |
| 1. I know how much money I spend on food in an average week | a. Strongly disagree  b. Disagree  c. Neutral  d. Agree  e. Strongly agree |
| 1. I often compare prices before I buy food | a. Strongly disagree  b. Disagree  c. Neutral  d. Agree  e. Strongly agree |
| 1. I try to get the best food for the best price | a. Strongly disagree  b. Disagree  c. Neutral  d. Agree  e. Strongly agree |
| 1. When food shopping, I compare prices between similar products in order to get the best value | a. Strongly disagree  b. Disagree  c. Neutral  d. Agree  e. Strongly agree |
| 1. When food shopping, I plan to take advantage of promotions | a. Strongly disagree  b. Disagree  c. Neutral  d. Agree  e. Strongly agree |
| 1. I often plan how much food to buy before I go shopping | a. Strongly disagree  b. Disagree  c. Neutral  d. Agree  e. Strongly agree |
| 1. Even if I don’t have my normal amount of time, I can still eat the food I prefer | a. Strongly disagree  b. Disagree  c. Neutral  d. Agree  e. Strongly agree |
| 1. Even if I don’t have my normal equipment, I can still eat the food I prefer | a. Strongly disagree  b. Disagree  c. Neutral  d. Agree  e. Strongly agree |
| 1. I use the time, money, skills and kitchen equipment I have to eat the food I prefer | a. Strongly disagree  b. Disagree  c. Neutral  d. Agree  e. Strongly agree |
| **Domain 2: Select (26 items)** | |
| **Component 2.1 Access food through multiple sources and know the advantages and disadvantages of these** | |
| 1. I try to purchase a variety of different types of food | a. Strongly disagree  b. Disagree  c. Neutral  d. Agree  e. Strongly agree |
| 1. I consider the environmental impact of the foods I eat | a. Strongly disagree  b. Disagree  c. Neutral  d. Agree  e. Strongly agree |
| 1. I consider the ethical impact of the foods I eat | a. Strongly disagree  b. Disagree  c. Neutral  d. Agree  e. Strongly agree |
| 1. When I’m eating out, it’s of great value to me that I can find the food I prefer | a. Strongly disagree  b. Disagree  c. Neutral  d. Agree  e. Strongly agree |
| 1. I find the foods I prefer to eat | a. Strongly disagree  b. Disagree  c. Neutral  d. Agree  e. Strongly agree |
| 1. When I’m in a new place, I find the foods I prefer to eat | a. Strongly disagree  b. Disagree  c. Neutral  d. Agree  e. Strongly agree |
| 1. I find the foods that align with my values | a. Strongly disagree  b. Disagree  c. Neutral  d. Agree  e. Strongly agree |
| 1. I find the foods I can afford | a. Strongly disagree  b. Disagree  c. Neutral  d. Agree  e. Strongly agree |
| 1. When I’m in a new place, I find the foods that align with my values | a. Strongly disagree  b. Disagree  c. Neutral  d. Agree  e. Strongly agree |
| **Component 2.2 Determine what is in a food product and where it came from** | |
| 1. I know where to look for information on what’s in fresh foods | a. Strongly disagree  b. Disagree  c. Neutral  d. Agree  e. Strongly agree |
| 1. The ingredients in packaged food products is important to me when deciding what foods to buy | a. Strongly disagree  b. Disagree  c. Neutral  d. Agree  e. Strongly agree |
| 1. I try to buy fresh food that is currently in season in my country | a. Strongly disagree  b. Disagree  c. Neutral  d. Agree  e. Strongly agree |
| 1. When eating out, I can make a judgement on what’s in the food I’ve selected | a. Strongly disagree  b. Disagree  c. Neutral  d. Agree  e. Strongly agree |
| 1. I know where to look for information on what’s in packaged foods | a. Strongly disagree  b. Disagree  c. Neutral  d. Agree  e. Strongly agree |
| 1. It’s easy to know what country different foods come from | a. Strongly disagree  b. Disagree  c. Neutral  d. Agree  e. Strongly agree |
| 1. I compare the kilojoules, fat, sugar or salt content on food products to guide what I buy | a. Strongly disagree  b. Disagree  c. Neutral  d. Agree  e. Strongly agree |
| 1. I know what’s in food that I buy if I’m food shopping | a. Strongly disagree  b. Disagree  c. Neutral  d. Agree  e. Strongly agree |
| 1. The social, environmental, economic impact of different foods influences what food I buy | a. Strongly disagree  b. Disagree  c. Neutral  d. Agree  e. Strongly agree |
| 1. I know what’s in food I could buy if I’m eating out* | a. Strongly disagree  b. Disagree  c. Neutral  d. Agree  e. Strongly agree |
| **Component 2.3 Judge the quality of food** | |
| 1. I can predict what fresh foods will be like before I buy it | a. Strongly disagree  b. Disagree  c. Neutral  d. Agree  e. Strongly agree |
| 1. I can predict what processed or convenience foods will be like before I buy it | a. Strongly disagree  b. Disagree  c. Neutral  d. Agree  e. Strongly agree |
| 1. I can predict what foods will be like before I buy it | a. Strongly disagree  b. Disagree  c. Neutral  d. Agree  e. Strongly agree |
| 1. I can predict what fresh food I’ve never come across before will be like before I buy it | a. Strongly disagree  b. Disagree  c. Neutral  d. Agree  e. Strongly agree |
| 1. I can predict what processed or convenience food I’ve never come across before will be like before I buy it | a. Strongly disagree  b. Disagree  c. Neutral  d. Agree  e. Strongly agree |
| 1. I can predict what foods I’ve never come across before will be like before I buy it | a. Strongly disagree  b. Disagree  c. Neutral  d. Agree  e. Strongly agree  f. Not sure/I don’t want to answer |
| 1. I read the expiry date information on food | a. Strongly disagree  b. Disagree  c. Neutral  d. Agree  e. Strongly agree |
| **Domain 3: Prepare (33 items)** | |
| **Component 3.1 Make a good tasting meal form whatever is available. This includes being able to prepare commonly available foods, efficiently use common pieces of kitchen equipment, and having sufficient repertoire of skills to adapt recipes (written or unwritten) to experiment with food and ingredients** | |
| 1. I am able to prepare and eat the food I prefer even if something unexpected happens in the short term | a. Strongly disagree  b. Disagree  c. Neutral  d. Agree  e. Strongly agree |
| 1. The food I eat on a typical day needs to be easy to prepare | a. Strongly disagree  b. Disagree  c. Neutral  d. Agree  e. Strongly agree |
| 1. I keep leftover food to eat at another time | a. Strongly disagree  b. Disagree  c. Neutral  d. Agree  e. Strongly agree |
| 1. I have the skills to prepare and cook affordable foods that I prefer | a. Strongly disagree  b. Disagree  c. Neutral  d. Agree  e. Strongly agree |
| 1. Knowing how to cook is of great value to me | a. Strongly disagree  b. Disagree  c. Neutral  d. Agree  e. Strongly agree |
| 1. I am able to cook from simple, staple ingredients | a. Strongly disagree  b. Disagree  c. Neutral  d. Agree  e. Strongly agree |
| 1. I can prepare a meal using fresh or minimally processed ingredients | a. Strongly disagree  b. Disagree  c. Neutral  d. Agree  e. Strongly agree |
| 1. When preparing food, I am confident about substituting alternative ingredients | a. Strongly disagree  b. Disagree  c. Neutral  d. Agree  e. Strongly agree |
| 1. I do not like to cook or prepare food | a. Strongly disagree  b. Disagree  c. Neutral  d. Agree  e. Strongly agree |
| I am confident…   1. Using a kitchen knife 2. Measuring out ingredients 3. Mixing and stirring foods 4. Peeling fruits and vegetables | a. Strongly disagree  b. Disagree  c. Neutral  d. Agree  e. Strongly agree |
| 1. I am able to prepare the food I prefer even if my living circumstances change | a. Strongly disagree  b. Disagree  c. Neutral  d. Agree  e. Strongly agree |
| 1. I know how to find information about preparing different foods | a. Strongly disagree  b. Disagree  c. Neutral  d. Agree  e. Strongly agree |
| 1. When preparing food, I know what to do when something goes wrong | a. Strongly disagree  b. Disagree  c. Neutral  d. Agree  e. Strongly agree |
| 1. I am confident preparing food from the ingredients I have on hand | a. Strongly disagree  b. Disagree  c. Neutral  d. Agree  e. Strongly agree |
| 1. When preparing food, I am confident about substituting with cheaper alternatives | a. Strongly disagree  b. Disagree  c. Neutral  d. Agree  e. Strongly agree |
| 1. I have the skills to prepare and cook the foods I prefer | a. Strongly disagree  b. Disagree  c. Neutral  d. Agree  e. Strongly agree |
| 1. I am able to prepare food that I prefer without a recipe | a. Strongly disagree  b. Disagree  c. Neutral  d. Agree  e. Strongly agree |
| **3.2 Apply basic principles of safe food hygiene and handling** | |
| 1. I wash or peel fruit and vegetables before eating them | a. Strongly disagree  b. Disagree  c. Neutral  d. Agree  e. Strongly agree |
| 1. After handling raw meat, poultry or fish, I wash my hands | a. Strongly disagree  b. Disagree  c. Neutral  d. Agree  e. Strongly agree |
| 1. Once I’ve thawed meat, I never refreeze it | a. Strongly disagree  b. Disagree  c. Neutral  d. Agree  e. Strongly agree |
| 1. I store raw meat separate from cooked meat | a. Strongly disagree  b. Disagree  c. Neutral  d. Agree  e. Strongly agree |
| 1. I use the same equipment for raw and cooked meat | a. Strongly disagree  b. Disagree  c. Neutral  d. Agree  e. Strongly agree |
| 1. I wipe down the kitchen surfaces before and after meals are prepared | a. Strongly disagree  b. Disagree  c. Neutral  d. Agree  e. Strongly agree |
| 1. I know how to store fruits and vegetables for best freshness and food safety | a. Strongly disagree  b. Disagree  c. Neutral  d. Agree  e. Strongly agree |
| 1. I always store meats and dairy at low temperatures | a. Strongly disagree  b. Disagree  c. Neutral  d. Agree  e. Strongly agree |
| 1. Before handling food, I wash my hands | a. Strongly disagree  b. Disagree  c. Neutral  d. Agree  e. Strongly agree |
| 1. I use the storage information instructions on packaged foods | a. Strongly disagree  b. Disagree  c. Neutral  d. Agree  e. Strongly agree |
| 1. I understand the storage and expiry information on packaged food | a. Strongly disagree  b. Disagree  c. Neutral  d. Agree  e. Strongly agree |
| 1. I use the storage and expiry date information on food when deciding whether to eat it | a. Strongly disagree  b. Disagree  c. Neutral  d. Agree  e. Strongly agree |
| 1. I measure the temperature of meat to see whether it is cooked enough | a. Strongly disagree  b. Disagree  c. Neutral  d. Agree  e. Strongly agree |
| **Domain 4: Eat (33 items)** | |
| **Component 4.1 Understanding food has an impact on personal wellbeing** | |
| 1. The type of food I eat influences my health | a. Strongly disagree  b. Disagree  c. Neutral  d. Agree  e. Strongly agree |
| 1. The type of food I eat influences my wellbeing | a. Strongly disagree  b. Disagree  c. Neutral  d. Agree  e. Strongly agree |
| 1. I know what foods to eat to keep me healthy | a. Strongly disagree  b. Disagree  c. Neutral  d. Agree  e. Strongly agree |
| 1. The type of food I eat influences whether I will experience particular illnesses | a. Strongly disagree  b. Disagree  c. Neutral  d. Agree  e. Strongly agree |
| 1. I understand what foods to eat to prevent diet related chronic disease (such as heart disease) | a. Strongly disagree  b. Disagree  c. Neutral  d. Agree  e. Strongly agree |
| 1. I am able to find reliable information on foods for the prevention or management of a disease or condition | a. Strongly disagree  b. Disagree  c. Neutral  d. Agree  e. Strongly agree |
| 1. I am able to use food/nutritional based dietary guidelines to improve my health | a. Strongly disagree  b. Disagree  c. Neutral  d. Agree  e. Strongly agree |
| **Component 4.2 Demonstrate self-awareness of the need to personally balance food intake. This influences knowing foods to include for good health, foods to restrict for good health and appropriate portion size and frequency** | |
| 1. It is easy to understand food and nutritional messages from government | a. Strongly disagree  b. Disagree  c. Neutral  d. Agree  e. Strongly agree |
| 1. The nutritional content of food products is important to me when deciding what foods to buy | a. Strongly disagree  b. Disagree  c. Neutral  d. Agree  e. Strongly agree |
| 1. I make a conscious effort to try and eat healthily | a. Strongly disagree  b. Disagree  c. Neutral  d. Agree  e. Strongly agree |
| 1. When deciding what to eat, I think about healthy choices | a. Strongly disagree  b. Disagree  c. Neutral  d. Agree  e. Strongly agree |
| 1. I use the nutritional label on food products to guide my purchases | a. Strongly disagree  b. Disagree  c. Neutral  d. Agree  e. Strongly agree |
| To maximise your health, should you enjoy or limit the following foods;   1. Vegetables 2. Fruits 3. Sugary foods and drinks 4. Wholegrains 5. Processed meats 6. Water 7. Milk, yoghurt, cheese 8. Foods with saturated fats 9. Foods with added salt | 1. Enjoy 2. Limit 3. Not sure/I don’t want to answer |
| 1. At certain times of the day, I get hungry because I am used to eating then | a. Strongly disagree  b. Disagree  c. Neutral  d. Agree  e. Strongly agree |
| 1. Sometimes when I start eating, I just can’t seem to stop | a. Strongly disagree  b. Disagree  c. Neutral  d. Agree  e. Strongly agree |
| 1. When I am with someone who is overeating, I normally overeat too | a. Strongly disagree  b. Disagree  c. Neutral  d. Agree  e. Strongly agree |
| **Component 4.3 Join in and eat in a social way** | |
| 1. I am comfortable eating with other people | a. Strongly disagree  b. Disagree  c. Neutral  d. Agree  e. Strongly agree |
| 1. Eating with other people is about more than just food | a. Strongly disagree  b. Disagree  c. Neutral  d. Agree  e. Strongly agree |
| 1. In my household, people often eat at different times | a. Strongly disagree  b. Disagree  c. Neutral  d. Agree  e. Strongly agree |
| 1. Eating brings people together in an enjoyable way | a. Strongly disagree  b. Disagree  c. Neutral  d. Agree  e. Strongly agree |
| 1. When eating with other people, it is of great value to me to eat at a table | a. Strongly disagree  b. Disagree  c. Neutral  d. Agree  e. Strongly agree |
| 1. I often eat together with other people | a. Strongly disagree  b. Disagree  c. Neutral  d. Agree  e. Strongly agree |
| 1. If I’m with other people, it is of great value to me to eat together | a. Strongly disagree  b. Disagree  c. Neutral  d. Agree  e. Strongly agree |
| 1. I eat while I’m going from place to place | a. Strongly disagree  b. Disagree  c. Neutral  d. Agree  e. Strongly agree |
| 1. I usually do other things while eating, such as reading, working, studying, using an electronic device or watching television | a. Strongly disagree  b. Disagree  c. Neutral  d. Agree  e. Strongly agree |
