# Supplementary material for: Progressing the development of a food literacy questionnaire using cognitive interviews
Source: Public Health Nutr. 2021 Nov 8;25(7):1968–78. doi: 10.1017/S1368980021004560 (PMC9991617; doi:10.1017/S1368980021004560)
Supplement: Supplementary file 1 [file S1368980021004560sup.zip › S1368980021004560sup002.docx]

# **Supplementary Material**

## Table S2 Food literacy questionnaires items were sourced from and whether face validity or cognitive interviews were conducted during the validation process

| **Reference** | **Number of participants** | **Cognitive interview, face validity method** |
| --- | --- | --- |
| Ailawadi, K. L., Neslin, S. A., & Gedenk, K. (2001). Pursuing the Value-Conscious Consumer: Store Brands versus National Brand Promotions. J Mark, 65(1), 71-89. | No questionnaire development/validation reported | |
| Amuta-Jimenez, A. O., Lo, C., Talwar, D., Khan, N., & Barry, A. E. (2019). Food label literacy and use among US adults diagnosed with cancer: Results from a national representative study. Journal of Cancer Education, 34(5), 1000-1009. | No questionnaire development/validation reported | |
| Barbour, L. R., Ho, M. Y. L., Davidson, Z. E., & Palermo, C. E. (2016). Challenges and opportunities for measuring the impact of a nutrition programme amongst young people at risk of food insecurity: A pilot study. Nutrition Bulletin, 41(2), 122-129. | No questionnaire development/validation reported | |
| Barton, K. L., Wrieden, W. L., & Anderson, A. S. (2011). Validity and reliability of a short questionnaire for assessing the impact of cooking skills interventions. Journal of Human Nutrition and Dietetics, 24(6), 588-595. | 20 | - 16 females, 4 males, 21-69 years - Individual discussions with adults residing in Scotland, typical of those attending cooking skill classes - Interviewed regarding ease of completion and comprehension + further probing if questions misunderstood |
| Bauer, K. W., Larson, N. I., Nelson, M. C., Story, M., & Neumark-Sztainer, D. (2009). Socio-environmental, personal and behavioural predictors of fast-food intake among adolescents. Public Health Nutrition, 12(10), 1767-1774. | No questionnaire development/validation reported | |
| Begley, A., Paynter, E., & Dhaliwal, S. S. (2018). Evaluation tool development for food literacy programs. Nutrients, 10(11), 1617. | 1012 (though not stated how many contributed to refinement of items) | - Assessed acceptability, comprehension of items - Program participants provided feedback on wording of questions, relevance of some food literacy behaviours |
| Bell, R., & Marshall, D. W. (2003). The construct of food involvement in behavioral research: scale development and validation. APPETITE, 40(3), 235-244. | 3 | - 1 experimenter, 2 psychologists - Rated for face validity on a 7-point scale (extremely low, extremely high) |
| Boucher, A. B., Elizabeth, M., Meaghan, R. B., Lynn, R., & Rebecca, T. (2017). The Ontario Food and Nutrition Strategy: identifying indicators of food access and food literacy for early monitoring of the food environment. Health promotion and chronic disease prevention in Canada: research, policy and practice, 37(9), 313. | No questionnaire development/validation reported | |
| Brissette, I., Lowenfels, A., Noble, C., & Spicer, D. (2013). Predictors of total calories purchased at fast-food restaurants: Restaurant characteristics, calorie awareness, and use of calorie information. J Nutr Educ Behav, 45(5), 404-411. | No questionnaire development/validation reported | |
| Buckley, M., Cowan, C., & McCarthy, M. (2007). The convenience food market in Great Britain: Convenience food lifestyle (CFL) segments. APPETITE, 49(3), 600-617. | Not described | |
| Burton, M., Reid, M., Worsley, A., & Mavondo, F. (2017). Food skills confidence and household gatekeepers' dietary practices. APPETITE, 108, 183-190. | Questionnaire developed, but cognitive interviews, face validity not described | |
| Byrd-Bredbenner, C. (2005). Food Preparation Knowledge and Confidence of Young Adults. Journal of Nutrition in Recipe & Menu Development, 3(3-4), 37- 50. | Questionnaire developed, but cognitive interviews, face validity not described | |
| Byrd-Bredbenner, C., Wheatley, V., Schaffner, D., Bruhn, C., Blalock, L., & Maurer, J. (2007). Development and implementation of a food safety knowledge instrument: Food science education research. Journal of Food Science Education, 6(3), 46-55. | Questionnaire developed, but cognitive interviews, face validity not described | |
| Cerjak, M., Haas, R., Brunner, F., & Tomic, M. (2014). What motivates consumers to buy traditional food products? Evidence from Croatia and Austria using word association and laddering interviews. Br Food J., 116(11), 1726-1747. | 59 | - 31 Croatian consumers, 28 Austrian consumers - Word association test (‘traditional food’) - Laddering interviews (bottom-up interview process starting with questions about lower-level attributes to uncover high-level values) |
| Chamhuri, N., & Batt, P. J. (2015). Consumer perceptions of food quality in Malaysia. Br Food J., 117(3), 1168-1187. | Not described | |
| Condrasky, M. D., Williams, J. E., Catalano, P. M., & Griffin, S. F. (2011). Development of psychosocial scales for evaluating the impact of a culinary nutrition education program on cooking and healthful eating. J Nutr Educ Behav, 43(6), 511-516. | 39 | - 3 focus groups (parents, caregivers, cooks) - Assisted in development of survey wording, appropriateness of questions, timing, readability |
| Crawford, D., Ball, K., Mishra, G., Salmon, J., & Timperio, A. (2007). Which food-related behaviours are associated with healthier intakes of fruits and vegetables among women? Public Health Nutrition, 10(3), 256-265. | Questionnaire developed, but cognitive interviews, face validity not described | |
| Dollahite, J. S., Pijai, E. I., Scott-Pierce, M., Parker, C., & Trochim, W. (2014). A Randomized Controlled Trial of a Community-Based Nutrition Education Program for Low-Income Parents. J Nutr Educ Behav, 46(2), 102-109. | No questionnaire development/validation reported | |
| Dunn, C., Jayaratne, K. S. U., Baughman, K., & Levine, K. (2014). Teaching Basic Cooking Skills: Evaluation of the North Carolina Extension "Cook Smart, Eat Smart" Program. J Fam Consum Sci, 106(1), 39-46. | No questionnaire development/validation reported | |
| Flego, A., Herbert, J., Gibbs, L., Swinburn, B., Keating, C., Waters, E., & Moodie, M. (2013). Methods for the evaluation of the Jamie Oliver Ministry of Food program, Australia. BMC Public Health, 13(1), 411. | 30 | - Jamie Oliver Ministry of Food population participants - Informal focus group sessions - Identification of questions that were ambiguous, sensitive |
| Food and Drug Administration. (2016). 2014 FDA Health and Diet Survey. | Questionnaire developed, but no validation described | |
| Garcia, A. L., Vargas, E., Lam, P. S., Shennan, D. B., Smith, F., & Parrett, A. (2014). Evaluation of a cooking skills programme in parents of young children - a longitudinal study. Public Health Nutrition, 17(5), 1013-1021. | Questionnaire developed, but cognitive interviews, face validity not described | |
| Grunert, K. G., Brunsø, K., & Bisp, S. (1993). Food-related life style: Development of a cross-culturally valid instrument for market surveillance. MAPP working paper no 12. | Not described | |
| Hartmann, C., Dohle, S., & Siegrist, M. (2013). Importance of cooking skills for balanced food choices. Appetite, 65, 125-131. | Questionnaire developed, but cognitive interviews, face validity not described | |
| Herbert, J., Flego, A., Gibbs, L., Waters, E., Swinburn, B., Reynolds, J., & Moodie, M. (2014). Wider impacts of a 10-week community cooking skills program - Jamie's Ministry of Food, Australia. BMC Public Health, 14(1), 1161. | No questionnaire development/validation reported | |
| Hutchinson, J., Watt, J. F., Strachan, E. K., & Cade, J. E. (2016). Evaluation of the effectiveness of the Ministry of Food cooking programme on self-reported food consumption and confidence with cooking. Public health nutrition, 19(18), 3417-3427. | No questionnaire development/validation reported | |
| Hwang, J., & Cranage, D. (2010). Customer health perceptions of selected fast-food restaurants according to their nutritional knowledge and health consciousness. Journal of Foodservice Business Research, 13(2), 68-84. | 30 | - Undergraduate students - Interviewed about understandability, readability of questionnaire |
| Jevšnik, M., Hlebec, V., & Raspor, P. (2008). Consumers’ awareness of food safety from shopping to eating. Food Control, 19(8), 737-745. | 20 | - Question clarity, identify response options, gauge duration |
| Kearney, J. M., Gibney, M. J., Livingstone, B. E., Robson, P. J., Kiely, M., & Harrington, K. (2001). Attitudes towards and beliefs about nutrition and health among a random sample of adults in the Republic of Ireland and Northern Ireland. Public Health Nutrition, 4(5a), 1117-1126. | No questionnaire development/validation reported | |
| Kliemann, N., Wardle, J., Johnson, F., & Croker, H. (2016). Reliability and validity of a revised version of the General Nutrition Knowledge Questionnaire. European Journal of Clinical Nutrition, 70(10), 1174-1180. | 20 | - 2 x dietitians, 3 x health psychologists, 15 x dietetics students - Assessed level of difficulty, adequacy of items, selected best items based on clarity, content, interpretability |
| Krause, C., Sommerhalder, K., Beer-Borst, S. (2016). Nutrition-specific health literacy: development and testing of a multi-dimensional questionnaire. Ernahrungs Umschau, 63, 214-220.  Krause, C. G., Beer-Borst, S., Sommerhalder, K., Hayoz, S., & Abel, T. (2018). A short food literacy questionnaire (SFLQ) for adults: Findings from a Swiss validation study. Appetite, 120, 275-280. | 13 | - 4 men, 9 women aged 27-67 - Thinking-aloud pre-test |
| Lahne, J., Wolfson, J. A., & Trubek, A. (2017). Development of the Cooking and Food Provisioning Action Scale (CAFPAS): A new measurement tool for individual cooking practice. Food Quality and Preference, 62, 96-105. | 7 | - Experts in food-studies (community nutritionists, chefs, food scientists, extension officers, rural sociologists) |
| Lavelle, F., McGowan, L., Hollywood, L., Surgenor, D., McCloat, A., Mooney, E., . . . Dean, M. (2017). The development and validation of measures to assess cooking skills and food skills. Int J Behav Nutr Phys Act., 14, 118. | 14 | - Pilot survey field interviews - Clarity of questions, how easy participants found measures to complete, wording, readability, usability |
| Levy, J., & Auld, G. (2004). Cooking Classes Outperform Cooking Demonstrations for College Sophomores. J Nutr Educ Behav, 36(4), 197-203. | Questionnaire developed and some initial validation but cognitive interviews, face validity not described | |
| Mallinson, L. J., Russell, J. M., & Barker, M. E. (2016). Attitudes and behaviour towards convenience food and food waste in the United Kingdom. APPETITE, 103, 17-28. | Questionnaire developed and some initial validation but cognitive interviews, face validity not described | |
| Méjean, C., Hassen, W. S., Gojard, S., Ducrot, P., Lampuré, A., Brug, H., ... & Castetbon, K. (2017). Social disparities in food preparation behaviours: a DEDIPAC study. Nutrition journal, 16(1), 1-13. | Not described  100 | - Experts (multidisciplinary research team of nutritionists, dietitians, economists, sociologists) assessed length, items’ wording, categories of responses - ‘Subjects’ evaluated acceptability, feasibility on 4-point likert scale strongly disagree to strongly agree for questionnaire length or redundant items |
| NatCen Social Research. (2008). British Social Attitudes Survey. | Questionnaire developed, but no validation described | |
| Neumark-Sztainer, D., Larson, N. I., Fulkerson, J. A., Eisenberg, M. E., & Story, M. (2010). Family meals and adolescents: what have we learned from Project EAT (Eating Among Teens)? Public Health Nutrition, 13(7), 1113-1121. | Questionnaire developed, but no validation described | |
| Palumbo, R., Annarumma, C., Adinolfi, P., Vezzosi, S., Troiano, E., Catinello, G., & Manna, R. (2017). Crafting and applying a tool to assess food literacy: Findings from a pilot study. Trends Food Sci Technol., 67, 173-182. | 15 | - 12 women, 3 men (26-58), members of the Italian Association of Dietitians - Provide feedback on design, clarity of the tool |
| Pinard, C. A., Uvena, L. M., Quam, J. B., Smith, T. M., & Yaroch, A. L. (2015). Development and testing of a revised Cooking Matters for Adults Survey. American Journal of Health Behavior, 39(6), 866-873. | 21 | - Think-aloud, probing |
| Poelman, M. P., Dijkstra, S. C., Sponselee, H., Kamphuis, C. B. M., Battjes-Fries, M. C. E., Gillebaart, M., & Seidell, J. C. (2018). Towards the measurement of food literacy with respect to healthy eating: the development and validation of the self perceived food literacy scale among an adult sample in the Netherlands. Int J Behav Nutr Phys Act, 15(1), 54. | Not described | |
| Ramirez, E. (2015). Development and implementation of the generations eating together through cooking (G.E.T.T. Cooking) curriculum and its effects on an inter-generational population: A pilot study. (Degree Doctor of Philosophy), Clemson University, Clemson, South Carolina, USA. | Questionnaire developed, but no validation described | |
| Sanlier, N., & Konaklioglu, E. (2012). Food safety knowledge, attitude and food handling practices of students. Br Food J, 114(4), 469-480. | Not described | - Face-to-face interviews (not further described) |
| Scholderer, J., Brunsø, K., Bredahl, L., & Grunert, K. G. (2004). Cross-cultural validity of the food-related lifestyles instrument (FRL) within Western Europe. APPETITE, 42(2), 197-211. |  |  |
| Steptoe, A., Pollard, T. M., & Wardle, J. (1995). Development of a Measure of the Motives Underlying the Selection of Food: the Food Choice Questionnaire. APPETITE, 25(3), 267-284. | Not described | |
| Stotts, J. L., & Lohse, B. (2007). Reliability of the ecSatter Inventory as a Tool to Measure Eating Competence. J Nutr Educ Behav, 39(5, Supplement), S167- S170. | Tested existing questionnaire and some initial validation but cognitive interviews, face validity not described | |
| The Health and Social Care Information Centre. (2009). Health survey for England 2007, Healthy lifestyles: Knowledge, attitudes and behaviour. | Questionnaire developed, but no validation described | |
| Thomas, H. M., & Irwin, J. D. (2011). Cook It Up! A community-based cooking program for at-risk youth: Overview of a food literacy intervention. BMC Research Notes, 4(1), 495. | No questionnaire development/validation reported | |
| Thonney, P. F., & Bisogni, C. A. (2006). Cooking Up Fun! A Youth Development Strategy that Promotes Independent Food Skills. J Nutr Educ Behav, 38(5), 321-323. | No questionnaire development/validation reported | |
| Vilaro, M. J., Zhou, W., Colby, S. E., Byrd-Bredbenner, C., Riggsbee, K., Olfert, M. D., . . . Mathews, A. E. (2017). Development and Preliminary Testing of the Food Choice Priorities Survey (FCPS): Assessing the Importance of Multiple Factors on College Students’ Food Choices. Evaluation & the Health Professions, 40(4), 425-449. | 12 | - 7 experts, 5 undergraduate students - Rated items for clarity, relevance, representativeness, coverage using yes/no rating system |
| Wallace, R., Lo, J., & Devine, A. (2016). Tailored nutrition education in the elderly can lead to sustained dietary behaviour change. The journal of nutrition, health & aging, 20(1), 8-15. | Questionnaire developed and some initial validation but cognitive interviews, face validity not described | |
| Wijayaratne, S. P., Reid, M., Westberg, K., Worsley, A., & Mavondo, F. (2018). Food literacy, healthy eating barriers and household diet. European Journal of Marketing. | Questionnaire developed and some initial validation but cognitive interviews, face validity not described | |
| Winkler, E., & Turrell, G. (2010). Confidence to Cook Vegetables and the Buying Habits of Australian Households. Journal of the American Dietetic Association, 110(5), S52-S61. | No questionnaire development/validation reported | |
| Woodruff, S. J., & Kirby, A. R. (2013). The associations among family meal frequency, food preparation frequency, self-efficacy for cooking, and food preparation techniques in children and adolescents. J Nutr Educ Behav, 45(4), 296-303. | 13 | - Children (not further described) - Cognitive interviews - Rewording of questions, options, additional items |
| Worsley, A., Wang, W. C., & Burton, M. (2015). Food concerns and support for environmental food policies and purchasing. APPETITE, 91, 48-55. | Questionnaire developed and some initial validation but cognitive interviews, face validity not described | |
| Ahn, S., Kim, B., Um, M., Park, Y., & Kye, S. (2020). Development and validation of a nutrition literacy assessment tool for young adults. Journal of Nutrition and Health, 53(2), 175-189. | Not in English | |
| Gibbs, H. D., Ellerbeck, E. F., Gajewski, B., Zhang, C., & Sullivan, D. K. (2018). The nutrition literacy assessment instrument is a valid and reliable measure of nutrition literacy in adults with chronic disease. Journal of nutrition education and behavior, 50(3), 247-257. | Questionnaire developed and some initial validation but cognitive interviews, face validity not described | |
| Kircher, K. A. (2018). Validity of an Instrument Developed that Measures the Home Food Environment and Food Literacy of Food Pantry Guests (Doctoral dissertation, University of Cincinnati). | 12 | - Cognitive interviews, probing questions |
| Kennedy, L. G., Kichler, E. J., Seabrook, J. A., Matthews, J. I., & Dworatzek, P. D. (2019). Validity and reliability of a food skills questionnaire. Journal of nutrition education and behavior, 51(7), 857-864. | 20 | - Undergraduate students - Interview to provide additional feedback, identify what they thought questionnaire was measuring |
| Hosaka, K. The Survey of Parent’s Food Literacy and Children’s OYATSU. | Not described | |
| Chambers, C. (2012). A pilot study: the use of a survey to assess the food knowledge of nutrition students at various levels of nutrition education. | Questionnaire developed and some initial validation but cognitive interviews, face validity not described | |
| Vanderkooy, P. (2010). Food skills of Waterloo Region adults. Fireside Chat Presentation. | No questionnaire development/validation reported | |
| Na, Y., & Cho, M. S. (2020). Development of a tool for food literacy assessment for young adults: findings from a Korean validation study. Asia Pacific Journal of Clinical Nutrition, 29(4), 876-882. | 15 | - Undergraduate nutrition or Masters/PhD in nutrition/food - Any elements of food literacy missing |
| Park, D., Park, Y. K., Park, C. Y., Choi, M. K., & Shin, M. J. (2020). Development of a Comprehensive Food Literacy Measurement Tool Integrating the Food System and Sustainability. Nutrients, 12(11), 3300. | 10 | - Adults (20-64) - Interviews to investigate if questions were ambiguous or too complex |
| Ringland, E. M., Gifford, J. A., Denyer, G. S., Thai, D., Franklin, J. L., Stevenson, M. M., ... & O'connor, H. T. (2016). Evaluation of an electronic tool to assess food label literacy in adult Australians: A pilot study. Nutrition & Dietetics, 73(5), 482-489. | Questionnaire developed and some initial validation but cognitive interviews, face validity not described | |
